# Causal effect of C-reaction protein and endometrial cancer: Genetic evidence of the role of inflammation in endometrial cancer

**DOI:** 10.1097/MD.0000000000040616

**Published:** 2024-11-22

**Authors:** Chenyang Zhao, Fei Chen, Qiong Li, Chen Tan, Wei Zhang, Lixiu Peng, Chaoyan Yue

**Affiliations:** a Department of Obstetrics and Gynecology, The First Affiliated Hospital of Jinan University, Guangzhou, China; b Department of Obstetrics and Gynecology, The First People’s Hospital of Chenzhou, Chenzhou, China; c Huadong Hospital, Fudan University, Shanghai, China; d Obstetrics and Gynecology Hospital of Fudan University, Shanghai, China.

**Keywords:** C-reaction protein, endometrial cancer, inflammation, Mendelian randomization

## Abstract

Consensus remains elusive regarding the relationship between C-reactive protein (CRP) levels and endometrial cancer (EC). Our study sought to elucidate the causal association between CRP and EC, aiming to contribute to the understanding of this complex interplay. We primarily utilized the random-effects inverse variance-weighted method. This approach served as the foundation for our analysis, complemented by 3 additional techniques, including Mendelian randomization-Egger, weighted-median, and weighted mode. A series of sensitivity analyses were also conducted to affirm the stability and reliability of our results. Employing the inverse variance-weighted method, our findings indicated that a one-unit increment in log-transformed CRP concentrations (mg/L) was associated with a relatively 9.7% increased risk of overall EC (odds ratio [OR] = 1.097, 95% confidence interval [CI]: 0.996–1.208, *P* = .061), an 11% higher risk of endometrioid endometrial cancer (OR = 1.110, 95% CI: 1.000–1.231, *P* = .049) and a 25% increased risk of non-endometrioid cancers (OR = 1.250, 95% CI: 1.005–1.555, *P* = .045). Sensitivity analyses did not reveal evidence of horizontal pleiotropy in the analysis of CRP and overall EC, endometrioid endometrial cancer, or non-endometrioid cancers (*P* > .05). In the reverse analysis, our data demonstrated that EC exert no reverse effect on CRP levels. Our study suggested causal relationships between CRP and an elevated risk of EC and its subtypes, which contribute to the ongoing discourse on the role of inflammation, as indicated by CRP levels, in the etiology of EC and its variants.

## 1. Introduction

Endometrial cancer (EC) stands as a significant global health concern, ranking among the foremost gynecological malignancies affecting women and accounting for a notable 4% of all cancer diagnoses in recent years.^[1]^ The International Agency for Research on Cancer projects a steep rise in EC incidence, estimating a more than 50% increase worldwide by 2040.^[2]^ Despite the majority of cases being diagnosed at an early stage, accounting for roughly 75% of all diagnoses, the lack of efficacious treatment options, particularly for elderly women who constitute about 73% of cases diagnosed beyond the age of 54,^[3]^ underscores the urgent need for improved understanding and management strategies. The etiology of EC remains enigmatic, hindering the development of effective prevention and treatment measures. Clinically and pathologically, EC can be categorized into 2 primary subtypes: endometrioid endometrial cancer (EEC), which predominantly affects younger women, and non-endometrioid cancer (NEC), characterized by their occurrence in older patients and poorer prognosis.^[4]^ These distinct subtypes may possess unique risk factors, with NEC presenting a particularly puzzling origin. The quest for biomarkers capable of enhancing risk stratification and facilitating earlier diagnosis, particularly for NEC, is of paramount importance.

Emerging evidence suggests that chronic inflammation plays a pivotal role in EC pathogenesis.^[5]^ C-reactive protein (CRP), a well-established marker of low-grade chronic inflammation, has been implicated in the increased risk of EC. Observational studies have consistently reported that elevated CRP concentrations, sustained for at least 12 months prior to cancer detection, correlate positively with EC incidence.^[6]^ A recent review corroborated the association between CRP and an increased EC risk.^[7]^ Another study revealed that elevated CRP levels are indeed associated with EC, but the risk increment was not substantial in diabetic patients, suggesting that the inflammatory intensity may vary among different cancer types and that the influence of CRP on EC might be attenuated in the presence of certain risk factors.^[8]^ While existing studies have established a link between high CRP concentrations and EC incidence, consensus on the nature of this relationship remains elusive, and a causal connection has yet to be firmly established. Case–control studies, often plagued by confounding factors such as age, body mass index (BMI), and comorbidities, struggle to control for these variables effectively. Moreover, the temporal relationship between exposure and outcome can introduce bias into causal estimations, making it challenging to ascertain a definitive causal relationship between CRP and EC.

Mendelian randomization (MR) represents a novel and powerful approach to elucidate causal associations between exposures and outcomes. By leveraging genetic variants as instrumental variables (IVs), MR enables researchers to estimate the causal relationships between exposures and outcomes.^[9]^ Given the random distribution of genotypes from parents to offspring, the association between genetic variants and outcomes is shielded from common confounders, akin to the randomization process in clinical trials.^[10]^ This methodology has gained traction in exploring the causal effects of various exposures on numerous diseases. In this study, we employ MR to investigate the causal effect of CRP on EC and its subtypes, aiming to bridge the gap in our understanding of this complex relationship and contribute to the development of targeted interventions for EC prevention and treatment. Through rigorous analysis and the utilization of advanced statistical methods, we endeavor to provide insights into the potential causal role of CRP in the etiology of EC, paving the way for future research and clinical applications.

## 2. Methods

### 2.1. Study design and data sources

**Figure 1.**
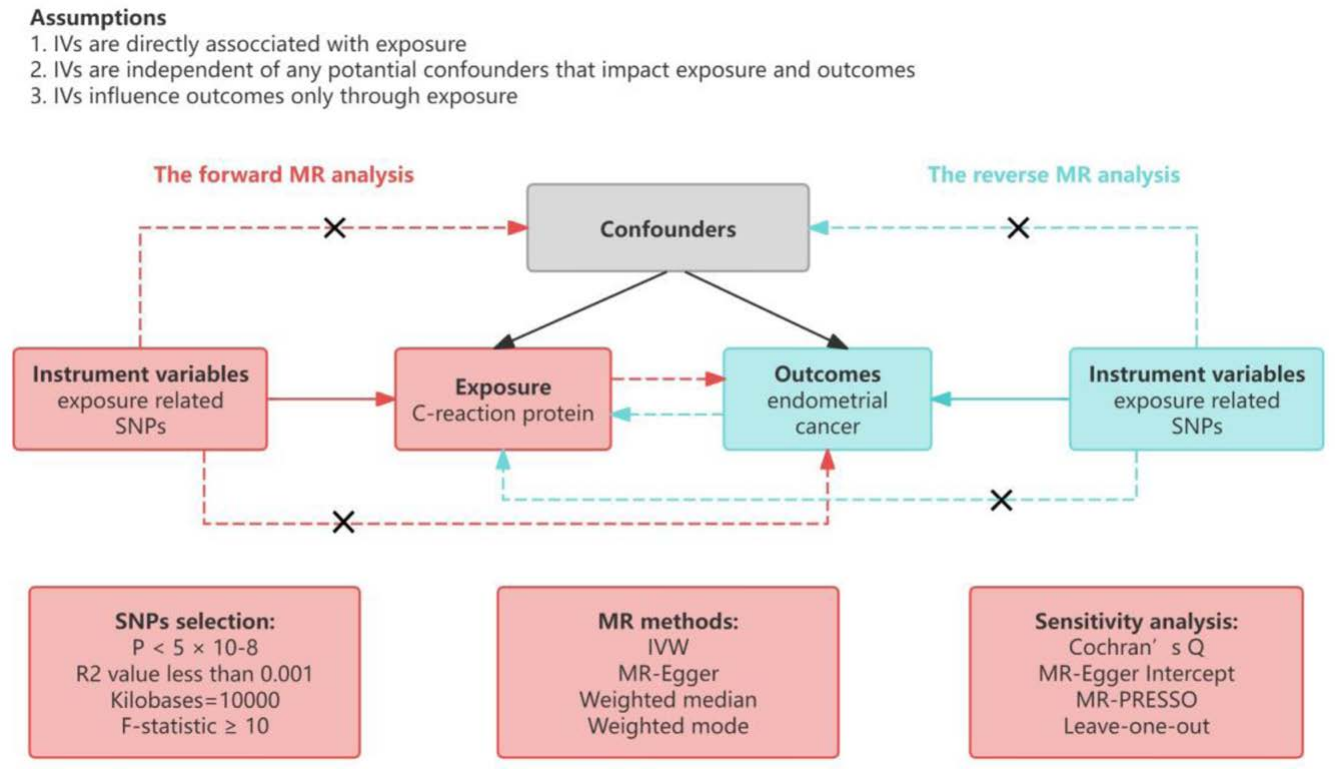
Graphical abstract for this MR study between C-reaction protein and overall endometrial cancer and its subtype endmetriod histolog and non-endmetrioid histology. IVW = inverse variance-weighted; MR = Mendelian randomization; SNPs = single nucleotide polymorphisms.

The primary objective of this investigation was to explore the potential causal associations between CRP levels and EC incidence through a two-sample MR analysis. A schematic representation of our study design is depicted in Figure 1. To uphold the integrity of our MR analysis, we ensured strict adherence to the 3 cardinal criteria: (i) the selected genetic variants exhibited a significant association with CRP levels, (ii) these variants lacked direct correlation with EC, and (iii) their influence on EC was mediated solely through their impact on CRP levels.^[11]^

Single nucleotide polymorphisms (SNPs) pertinent to both CRP and EC were sourced from the Integrative Epidemiology Unit Open Genome-wide Association Study Project, a comprehensive repository of genetic associations. The CRP-related dataset encompassed a vast cohort of 353,466 individuals of European descent, featuring 19,057,467 SNPs. Detailed information pertaining to this dataset has been previously documented^[12]^ and the demographic profile of the study participants is outlined in Table S1, Supplemental Digital Content, http://links.lww.com/MD/N986. The EC-related data stemmed from a meta-analysis conducted by O’Mara et al comprising summary-level statistics from 12,906 cases and 108,979 controls of European ancestry.^[13]^ To enhance the specificity of our analysis, EC cases were stratified into 2 subtypes based on histology: EEC and NEC, with 8758 and 1230 cases, respectively. Comprehensive descriptions of these studies have been previously reported. Given the utilization of publicly accessible summary data for all analyses within our study, ethical approval was not required.

### 2.2. Selection of the genetic instruments

The SNPs that displayed a robust association with CRP concentration were selected, adhering to a stringent genome-wide significance threshold of *P* < 5 × 10^-8^. To ensure independence among the selected SNPs, we excluded those in linkage disequilibrium with others, defined by an *r*^2^ value <0.001 and a physical distance exceeding 10,000 kilobases. A further refinement involved the elimination of SNPs strongly correlated with the outcome. Initially, 199 independent SNPs were identified as potential IVs associated with CRP. Following harmonization procedures and the exclusion of palindromic SNPs with intermediate allele frequencies, the final tally stood at 188 IVs for overall EC, 182 for EEC, and 181 for NEC. To ascertain the adequacy of these IVs in explaining CRP variability, we calculated the F-statistic using the formula: F-statistic = *R*² × (SampleSize − 2)/(1 − *R*²).^[14]^ All the IVs selected for this study met the criterion of F-statistic ≥ 10, affirming their suitability as strong IVs. Furthermore, employing identical methods, we selected SNPs associated with EC for conducting reverse MR analyses, ensuring a consistent and rigorous approach throughout our investigation.

### 2.3. Statistical analysis

We undertook a comprehensive evaluation of the association between CRP and the risk of overall and subtype-specific EC. Our primary analytical strategy involved the use of the random-effects inverse variance-weighted (IVW) method to estimate the overall causal effect of the exposure on the outcome, which is renowned for providing the most accurate causal estimation.^[15]^ The causal effect of exposure on the outcome was quantified by calculating odds ratios (ORs) and corresponding 95% confidence intervals (CIs). To reinforce the robustness of our IVW estimate, we also employed 3 complementary methods: MR-Egger, weighted-median, and weighted mode. These additional techniques served to cross-validate our findings and ensure the consistency of the causal inference.

Moreover, a suite of sensitivity analyses was conducted to ascertain that the IVs affected the outcome solely through their impact on the exposure. This was achieved through the implementation of IVW, MR-Egger, MR-Egger Intercept, and MR-Pleiotropy Residual Sum and Outlier (MR-PRESSO) Global Test. The IVW method gauged heterogeneity between causal estimates across all SNPs by computing Cochran Q statistic, lending greater credibility to the causal effect. MR-Egger, another tool for assessing heterogeneity, also utilized Cochran Q test. Evidence of heterogeneity was indicated by a Cochran Q statistic *P*-value below .05. The MR-Egger Intercept, a method adept at identifying small-study reporting bias in meta-analyses, was utilized to detect bias stemming from pleiotropic effects. Additionally, we employed the MR-PRESSO method to identify and rectify horizontal pleiotropy and potential outliers.^[16]^ Horizontal pleiotropy was deemed present when the intercept of the MR-Egger regression significantly deviated from zero (*P* < .05) or when asymmetry in the funnel plot was observed. Furthermore, a leave-one-out sensitivity analysis was performed to evaluate the reliability of the causal estimates derived from the IVW method. This was accomplished by sequentially excluding each SNP from the analysis and recalculating the causal effect to ensure the stability of our findings.

All statistical analyses were conducted utilizing the TwoSampleMR and MR-PRESSO packages integrated into the R software environment, version 4.3.0. Strict adherence to the STROBE-MR statement guidelines was maintained throughout the study to ensure transparent and comprehensive reporting of our MR findings in the field of epidemiology.^[17]^

## 3. Results

After a thorough screening process, we identified 188 independent SNPs as genetic instruments for CRP, with their detailed characteristics and information compiled in Table S2, Supplemental Digital Content, http://links.lww.com/MD/N986. Among these, 6 SNPs (rs2306881, rs28429148, rs35308741, rs35440896, rs7662792, and rs832578) were excluded from the analyses of EEC and 7 SNPs (rs10831676, rs11644809, rs12713422, rs178795, rs35308741, rs45582641, and rs77704739) were excluded from the NEC due to potential weak instrument bias. Notably, all genetic instruments retained in our study exhibited an F-statistic surpassing the critical threshold of 10, ensuring their suitability for MR analyses.

**Figure 2.**
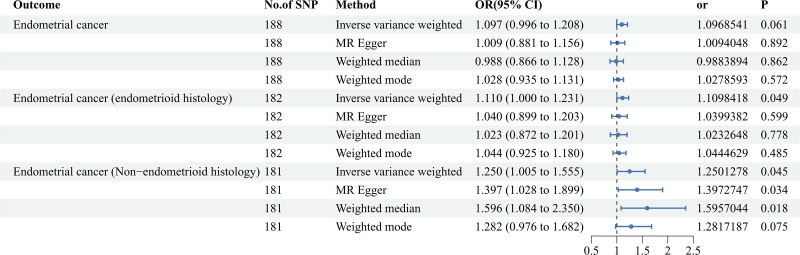
Forest plot to visualize the casual effect of C-reaction protein on overall endometrial cancer and its subtypes endmetriod histolog and non-endmetrioid histology in the forward MR analysis. Four methods including inverse variance-weighted, MR-Egger, weighted-median and weighted mode were conducted for MR analyses. CI = confidence interval; MR = Mendelian randomization; NO = number; OR = odds ratio; SNP = single nucleotide polymorphism.

Our primary analysis utilizing the IVW method revealed that a one-unit increase in log-transformed CRP concentrations (mg/L) was associated with a 9.7% heightened risk of overall EC, although the association did not reach statistical significance (OR = 1.097, 95% CI: 0.996–1.208, *P *= .061). This finding was consistent across alternative MR methods. However, subgroup analyses by histological subtype yielded intriguing results: a one-unit increase in log-transformed CRP concentration corresponded to an 11% increased risk of EEC (OR = 1.110, 95% CI: 1.000–1.231, *P* = .049) and a 25% increased risk of NEC (OR = 1.250, 95% CI: 1.005–1.555, *P* = .045) when applying the IVW method. The weighted-median, weighted mode, and MR-Egger methods corroborated these findings, presenting qualitatively similar outcomes (Fig. 2 and Table S3, Supplemental Digital Content, http://links.lww.com/MD/N986). The scatter plot to visualize casual effect of C-reaction protein on the risk overall EC, EEC, and NEC were presented in Figure S1, Supplemental Digital Content, http://links.lww.com/MD/N986. In sensitivity analyses, we employed 4 methods, including IVW, MR-Egger, MR-Egger Intercept, and MR-PRESSO Global Test, to scrutinize potential violations of MR assumptions. The IVW and MR-Egger tests suggested evidence of heterogeneity in the analyses of CRP and overall EC, as well as EEC (*P* < .05). Conversely, no heterogeneity was detected in the analysis of CRP and NEC (*P* > .05). The MR-Egger intercept test did not indicate any evidence of horizontal pleiotropy (*P* > .05), a conclusion more reliable than that derived from the MR-PRESSO Global Test. Consistently, neither method detected horizontal pleiotropy in the analysis of CRP and NEC (*P* > .05) (Table 1). The funnel plots to visualize the heterogeneity of MR estimates for the effect of C-reaction protein on the risk of overall EC, EEC, and NEC were showed in Figure S2, Supplemental Digital Content, http://links.lww.com/MD/N986. Our leave-one-out sensitivity analysis confirmed the absence of an outlier SNP whose variant-specific causal estimate markedly differed from those of other SNPs (Figures S3–S5, Supplemental Digital Content, http://links.lww.com/MD/N986).

**Table 1:** Association of C-reaction protein with endometrial cancer in sensitivity analysis.

Outcome	Heterogeneity				Pleiotropy
	Cochran Q of IVW	*P*-value of Q test	Cochran Q of MR-Egger	*P*-value of Q test	*P*-value of Egger Intercept
Endometrial cancer	314.71	<.001	309.85	<.001	.09
Endometrial histolog	249.32	<.001	247.19	<.001	.22
Nonendometrial histolog	173.88	.61	172.87	.62	.32

IVW = inverse variance-weighted, MR = Mendelian randomization.

**Figure 3.**
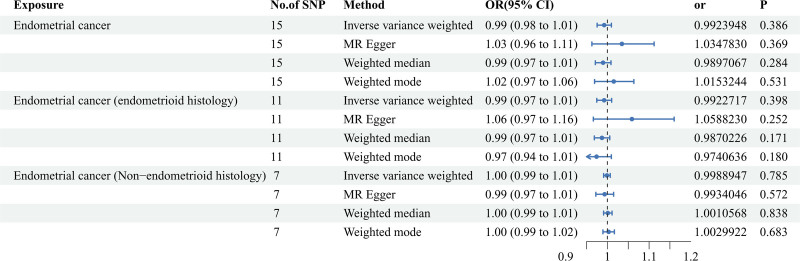
Forest plot to visualize the causal effect of overall endometrial cancer and its subtypes endmetriod histolog and non-endmetrioid histology on C-reaction protein in the reverse MR analysis. Four methods including inverse variance-weighted, MR-Egger, weighted-median, and weighted mode were conducted for MR analyses. CI = confidence interval; MR = Mendelian randomization; NO = number; OR = odds ratio; SNP = single nucleotide polymorphism.

In the reverse MR study, the results collectively refuted the hypothesis that EC might exert a reverse effect on CRP levels. All 4 MR methods, including IVW, MR-Egger, weighted-median, and weighted mode, yielded consistent findings. Moreover, the results demonstrated that the subtypes of EC exerted no significant influence on CRP levels (Fig. 3 and Table S4, Supplemental Digital Content, http://links.lww.com/MD/N986). The scatter plots and funnel plots were presented in Figure S6, Supplemental Digital Content, http://links.lww.com/MD/N986 and Figure S7, Supplemental Digital Content, http://links.lww.com/MD/N986. The leave-one-out sensitivity analysis of the reverse MR analyses was showed in Figures S8–S10, Supplemental Digital Content, http://links.lww.com/MD/N986.

## 4. Discussion

In this study, we first used a two-sample MR analyses to evaluate the effects of CRP on the risk of EC and its subtypes. Our results demonstrate that CRP is causally related to the risk of EC and its subtypes. Although there was no statistical difference in the MR ananlyses between CRP and overall EC (*P* > .05), subgroup analyses according to histotype indicated that high concentration of CRP increased the risk of EEC and NEC. Furthermore, one-unit increase in the log-transformed CRP concentration was associated with a 25% higher risk of NEC which proved CRP has a more significant causal effect in NEC than EEC. As cancer progression itself could lead to cancer-related inflammation and elevated concentrations of circulating pro-inflammatory biomarkers. We also performed reverse MR analyses and we did not find any casual effect of EC and its subtypes on CRP.

Numerous observational studies have previously explored the relationship between CRP and EC. Wang LJ and colleagues^[6]^ demonstrated that high CRP concentrations are associated with a higher incidence of EC. Another prospective study underscored the association between relatively high CRP levels and an increased EC risk, distinctively noting that this correlation was not observed for other inflammatory markers such as IL-6 or tumor necrosis factor-alpha (TNF-α). As a pro-inflammatory cytokine, CRP has been implicated in EC development through its role in modulating insulin and estradiol levels.^[18]^ However, a prospective case–control study noted that the association between CRP and EC, though present in univariable analysis, was significantly diminished and became nonsignificant upon adjusting for BMI.^[19]^ Few studies have delved into the relationship between CRP and the risk of EC subtypes. Recognizing the existence of 2 primary histological subtypes of EC, prior research from a clinical trial suggested a connection between CRP and the development of EEC, along with its impact on survival.^[20]^ A population-based case–control study investigating EC assessed the correlation between inflammatory mediators, including TNF-α, IL-6, and CRP, and the risk of EC and its subtypes. Their results revealed that elevated mean levels of these inflammatory mediators were significantly associated with an increased risk for EC and its subtypes. After multivariable adjustments, the association of CRP remained statistically significant (OR = 1.22, 95% CI: 1.02–1.47). Comparatively, CRP appears to have a stronger association with EEC (OR = 1.25, 95% CI: 1.03–1.52) than with NEC.^[21]^ Our study similarly elucidates the potential relationship between CRP and EC, albeit with a contrasting finding: the causal effect appears more pronounced in the NEC group than in the EEC group. The discrepancies observed across previous studies compared to ours can be attributed to various factors. Prior research may have been subject to biases from confounding variables such as age, BMI, and duration of observation. Study designs might have been confined to specific populations, such as premenopausal or postmenopausal women. Our MR analysis, free from such confounding factors, provides a more objective perspective on the relationship between CRP and EC. We are among the first to employ MR analysis to investigate the association between CRP and EC subtypes, and our results suggest that the impact of inflammatory biomarkers on EC risk varies by cancer type. Given that NEC is a less common yet more aggressive form of EC, its etiology may diverge from the more prevalent EEC and may be more heavily influenced by inflammatory processes.

Indeed, the intricate biological mechanisms by which inflammatory biomarkers escalate the risk of EC have been subjects of extensive research over the years. Inflammation is now recognized as a key player in cancer development, actively participating in its pathogenesis and accelerating tumor progression.^[22]^ Obesity, a significant contributor to EC etiology, is characterized by a persistent low-grade inflammatory condition.^[23,24]^ Pro-inflammatory cytokines such as IL-1, IL-6, TNF-α, and interferon-gamma are implicated in tumorigenesis by causing DNA damage, stimulating angiogenesis, and enhancing pro-proliferative and anti-apoptotic processes.^[25,26]^ CRP, classified as an acute-phase protein predominantly synthesized in the liver in response to IL-6 stimulation,^[27]^ emerges as a critical factor. Research by Salaroglio et al^[28]^ elucidates that CRP can drive cell proliferation and migration, influence apoptosis, cell differentiation, and senescence by activating the MAPK/ERK signaling pathway. Another intriguing mechanism by which CRP contributes to carcinogenesis involves the upregulation of low-density lipoprotein receptor-1 (LOX-1), which has been shown to facilitate cancer cell proliferation, angiogenesis, and metastasis.^[29]^ Furthermore, CRP may foster EC development and progression through alterations in autophagy. Ma et al’s study on clear cell renal cell carcinoma reveals a strong correlation between CRP levels and the expression of autophagy-related protein 9B (ATG9B), a regulator of the autophagy process that, when upregulated, promotes cancer cell survival and proliferation.^[30]^ These findings collectively underscore the pivotal role of CRP in EC development. However, it is crucial to acknowledge that cancer mechanisms should be evaluated within the context of specific subtypes. Our study highlights that the impact of CRP seems to be subtype-dependent, suggesting that the pathogenesis of different EC subtypes warrants further exploration. Future research should aim to unravel the unique mechanisms underlying each EC subtype to gain a comprehensive understanding of the disease’s heterogeneity.

Our study represents a groundbreaking achievement in elucidating the causal link between CRP and EC, particularly NEC. This discovery enhances our understanding of EC etiology and holds significant potential for refining preventive strategies, particularly for NEC, where early detection and intervention are paramount. The clinical utility of CRP as a biomarker is underscored by its simplicity, low cost, and ease of measurement. Elevated CRP levels in blood samples can serve as a red flag, guiding the targeted monitoring and management of women at heightened risk of EC, complemented by additional risk factor assessments.

Our investigation boasts several methodological strengths. As the inaugural study to apply MR to examine circulating CRP in the context of EC, we leveraged a substantial sample size sourced from publicly available databases, ensuring robust statistical power. This approach enabled us to confidently establish an association between CRP and EC. We further fortified our findings by conducting reverse MR analyses, ruling out any significant association between EC and CRP, thus negating reverse causality. Through meticulous adherence to the 3 core assumptions of MR and a battery of sensitivity tests, our study minimized confounding influences, demonstrating an advantage over randomized controlled trials. Additionally, our focus on stratifying outcomes by EC histotype addressed a critical oversight in prior observational studies. Notwithstanding these strengths, our study is not without limitations. Firstly, while the OR for overall EC based on CRP levels exceeds 1, the lack of statistical significance may stem from a relatively small sample size. Despite this, the observed trend supports a positive effect of CRP on overall EC risk. Secondly, the possibility of residual pleiotropy, a common concern in MR studies, cannot be entirely discounted; however, our comprehensive sensitivity analyses mitigate this issue, maintaining the credibility of our results. Lastly, the restriction of our study sample to individuals of European ancestry limits the generalizability of our findings to other racial and ethnic groups.

## 5. Conclusion

In summary, our bidirectional MR analyses provide compelling evidence for the causal role of physiological CRP concentrations in elevating the risk of EC and its subtypes, notably NEC. Independent of confounding variables and mediators, our findings offer genetic insights that can inform risk stratification strategies for EC, marking a significant step forward in personalized medicine and cancer prevention.

## Acknowledgments

We thank all the participants in this study. We also thank the IUE summary database for providing data for the analyses. Thanks for the financial support from the Public Data Pool of Medical Collaboration between the First People’s Hospital of Chenzhou and the Tenth People’s Hospital of Shanghai.

## Author contributions

**Conceptualization:** Chenyang Zhao, Fei Chen, Qiong Li, Lixiu Peng, Chaoyan Yue.

**Data curation:** Chenyang Zhao, Qiong Li, Chen Tan, Wei Zhang, Lixiu Peng, Chaoyan Yue.

**Formal analysis:** Chenyang Zhao, Fei Chen, Qiong Li, Lixiu Peng, Chaoyan Yue.

**Investigation:** Chenyang Zhao, Chaoyan Yue.

**Methodology:** Chenyang Zhao, Qiong Li, Lixiu Peng, Chaoyan Yue.

**Project administration:** Lixiu Peng, Chaoyan Yue.

**Resources:** Chenyang Zhao, Chaoyan Yue.

**Supervision:** Lixiu Peng, Chaoyan Yue.

**Software:** Chenyang Zhao, Chaoyan Yue.

**Validation:** Chenyang Zhao, Fei Chen, Qiong Li, Chen Tan, Wei Zhang, Lixiu Peng, Chaoyan Yue.

**Visualization:** Chenyang Zhao, Lixiu Peng, Chaoyan Yue.

**Writing – original draft:** Chenyang Zhao, Fei Chen, Qiong Li.

**Writing – review & editing:** Chenyang Zhao, Fei Chen, Qiong Li, Chen Tan, Wei Zhang, Lixiu Peng, Chaoyan Yue.

## Supplementary Material


