# Supplementary material for: Causal effect of C-reaction protein and endometrial cancer: Genetic evidence of the role of inflammation in endometrial cancer
Source: Medicine (Baltimore). 2024 Nov 22;103(47):e40616. doi: 10.1097/MD.0000000000040616 (PMC11596508; doi:10.1097/MD.0000000000040616)
Supplement: Supplementary file 1 [file medi-103-e40616-s001.pdf]

Supplemetary Figure legends

Supplementary Figure S1 Scatter plot to visualize casual effect of C-reaction protein on the risk of endometrial cancer and its subtypes

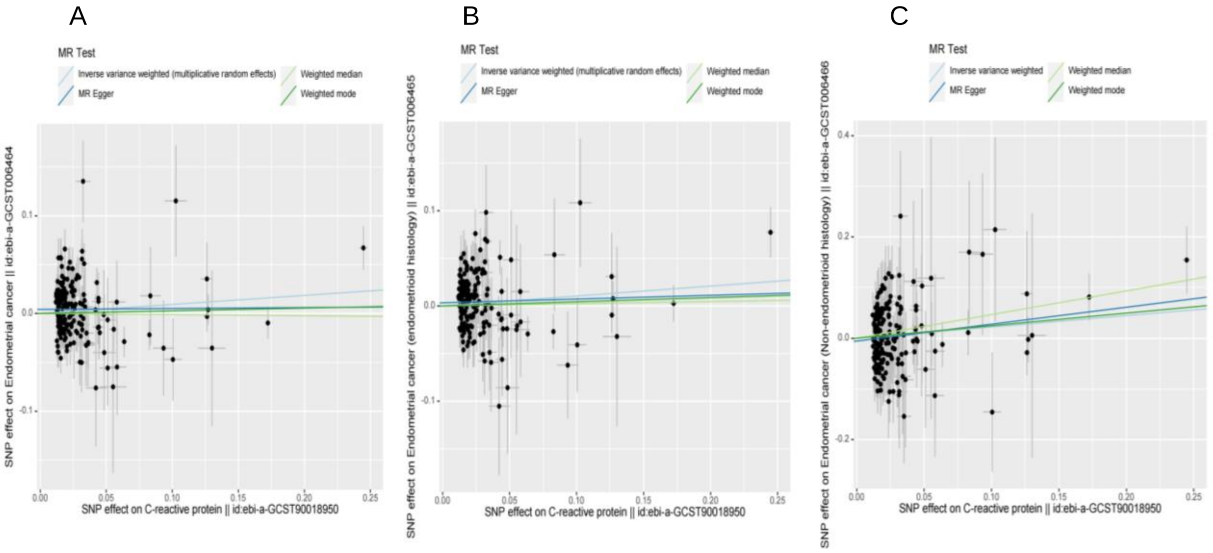

Supplementary Figure S2 Funnel plots to visualize the heterogeneity of MR estimates for the effect of C-reaction protein on the risk of endometrial cancer and its subtypes

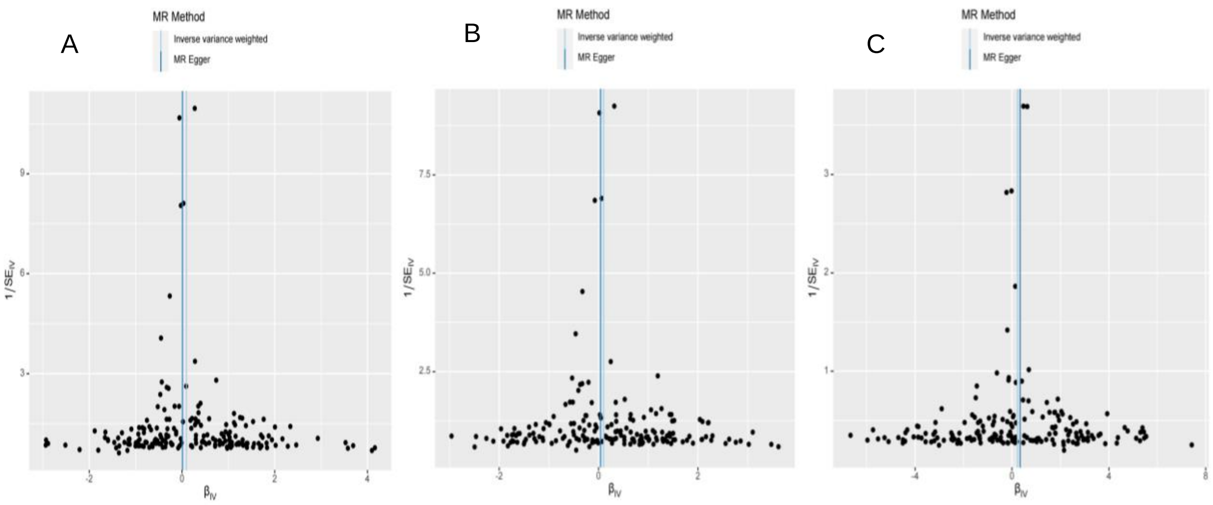

Supplementary Figure S3 Leave-one-out inverse-variance weighted mendelian randomization analysis of C-reaction protein on the risk of everall endometrial cancer

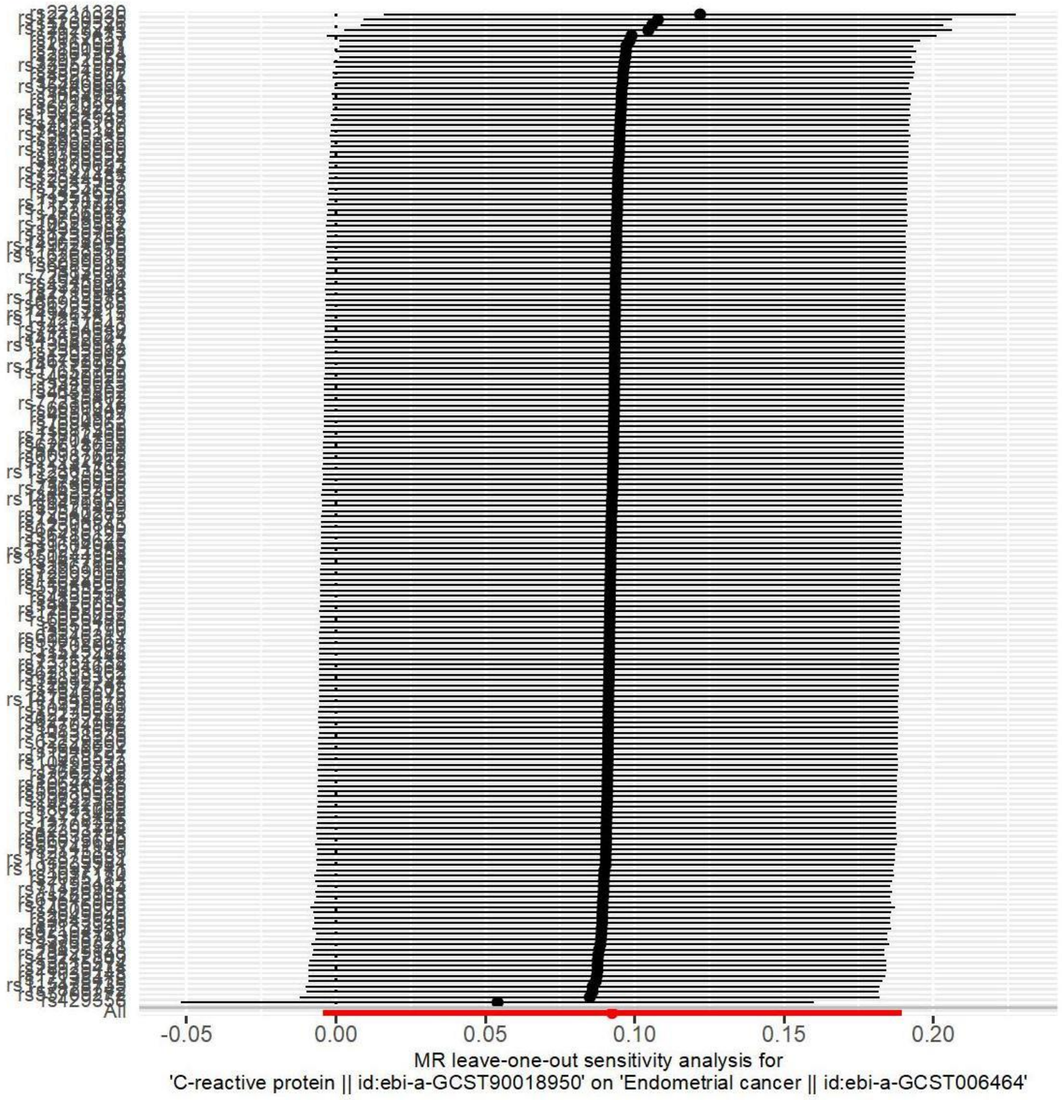

Supplementary Figure S4 Leave-one-out inverse-variance weighted mendelian randomization analysis of C-reaction protein on the risk of endometrioid histology cancer

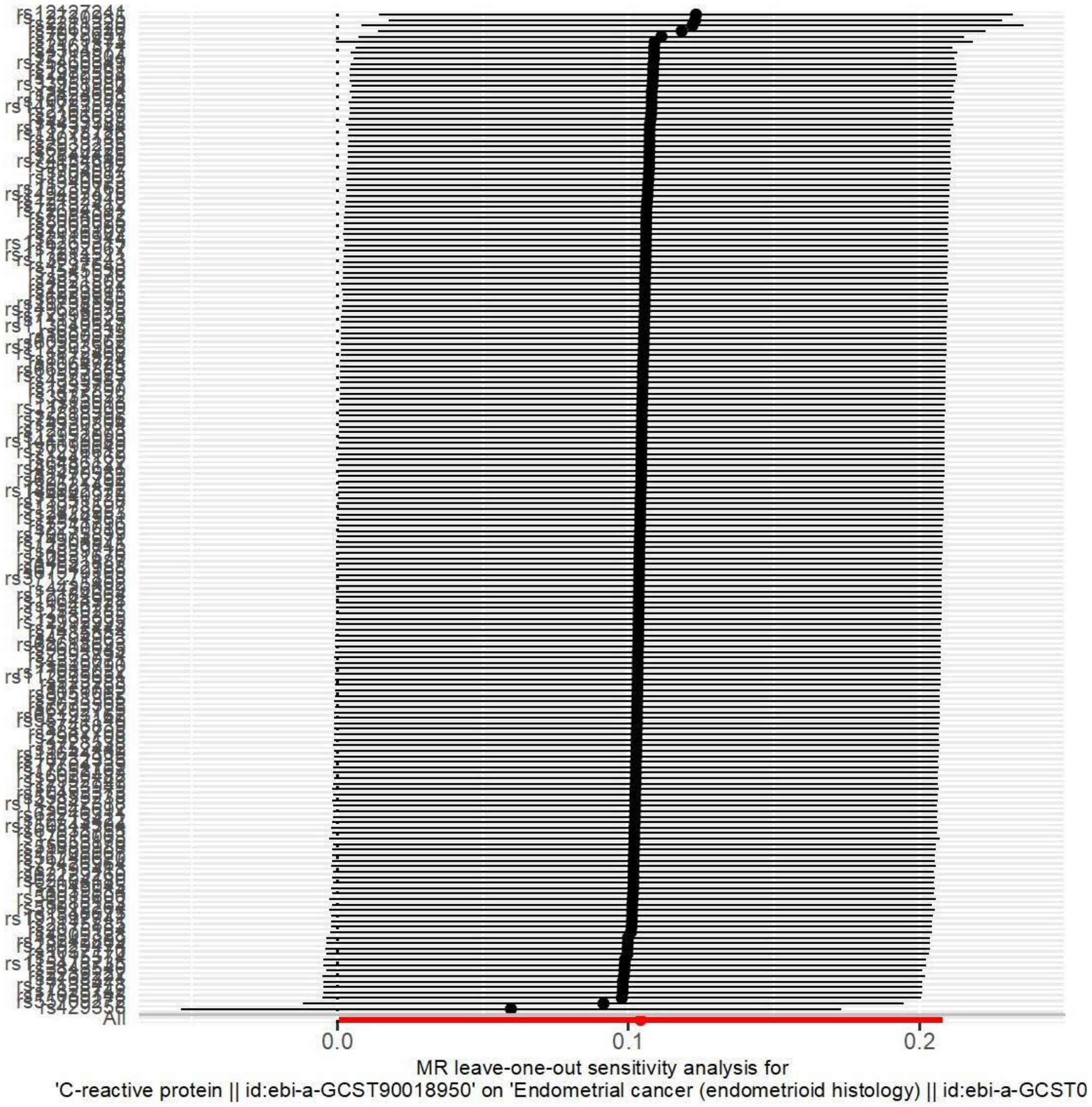

Supplementary Figure S5 Leave-one-out inverse-variance weighted mendelian randomization analysis of C-reaction protein on the risk of non-endometrioid histology cancer

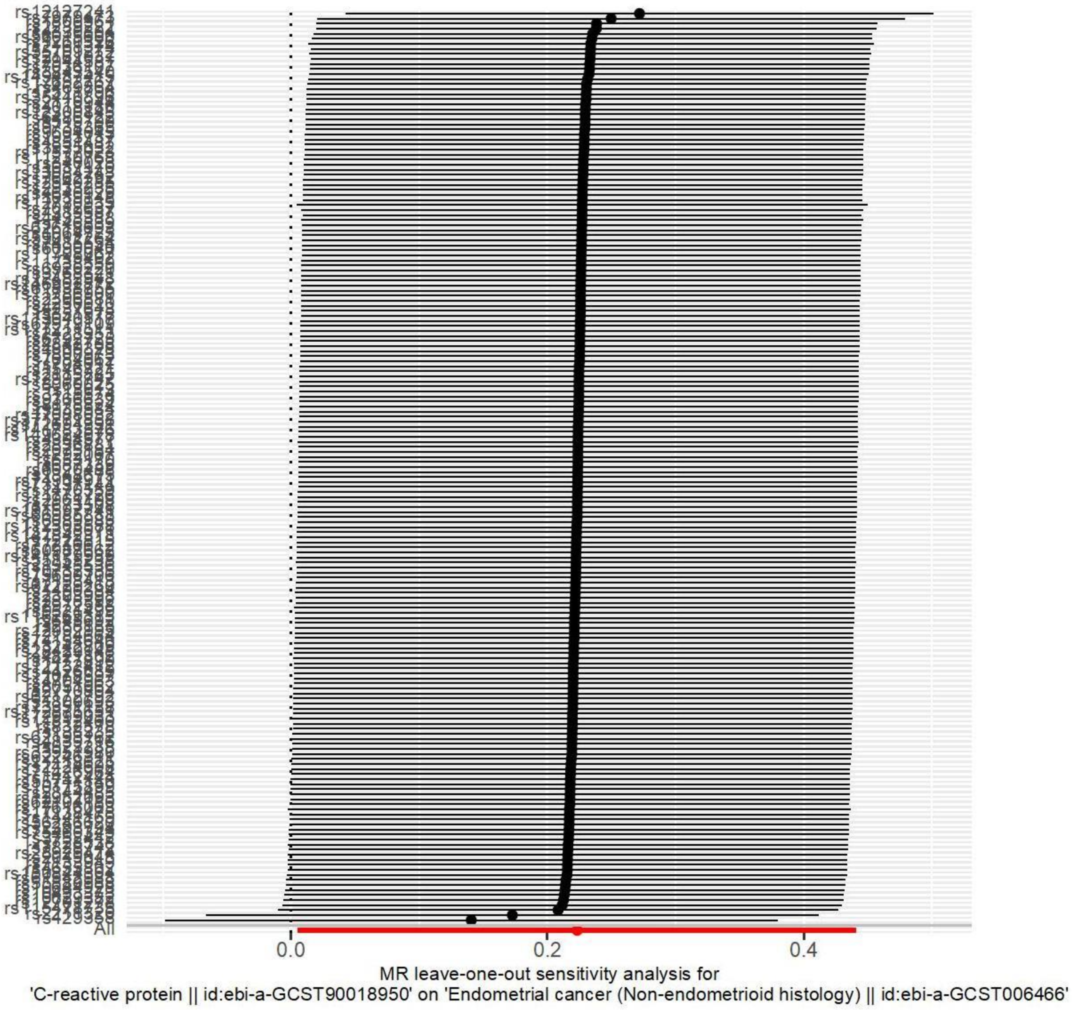

Supplementary Figure S6 Scatter plot to visualize casual associations between endometrial cancer and its subtypes and C-reaction protein

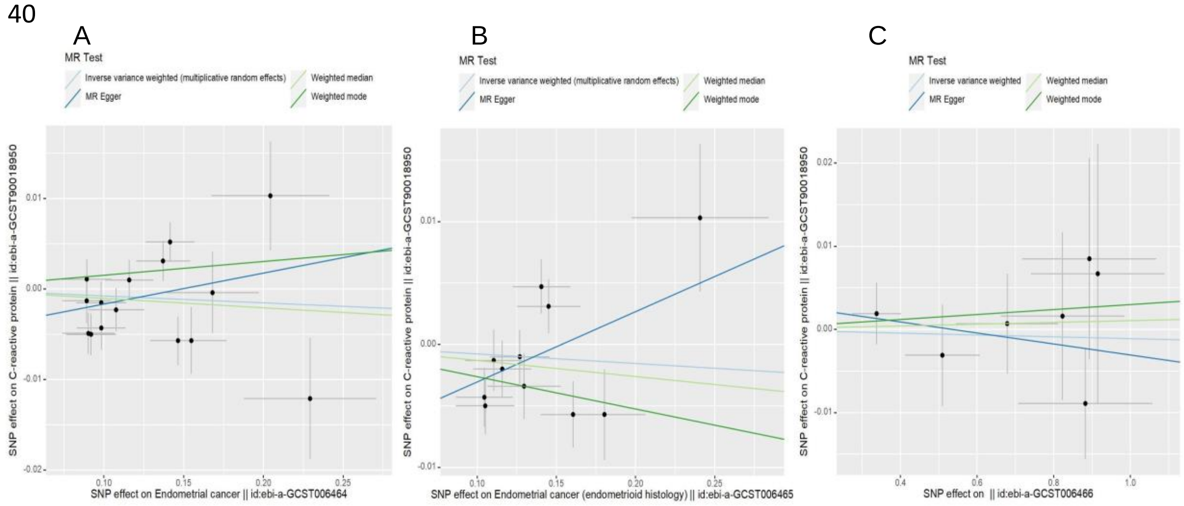

Supplementary Figure S7 Funnel plots to visualize the heterogeneity of MR estimates for the effect of endometrial cancer and its subtypes on C-reaction

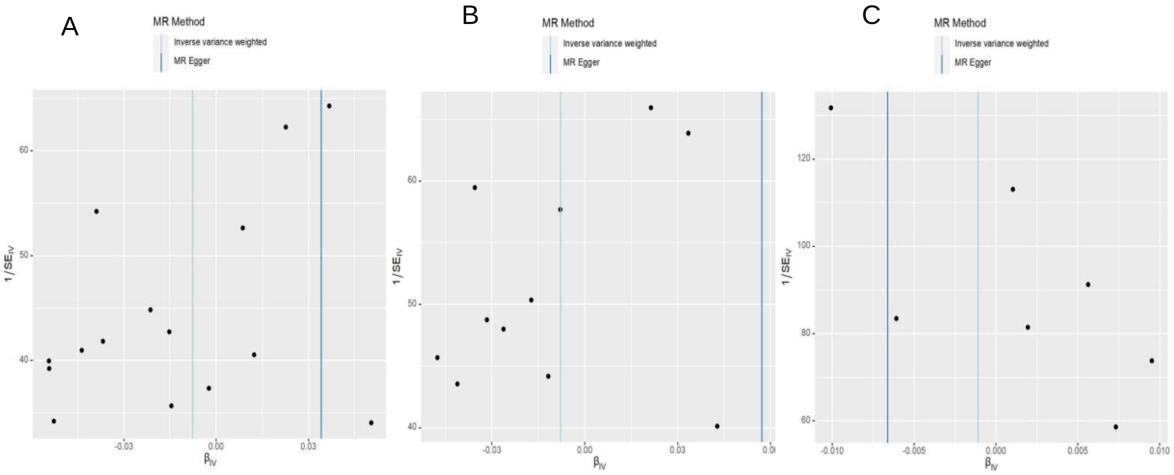

Supplementary Figure S8 Leave-one-out inverse-variance weighted mendelian randomization analysis of overall endometrial cancer on C-reaction protein

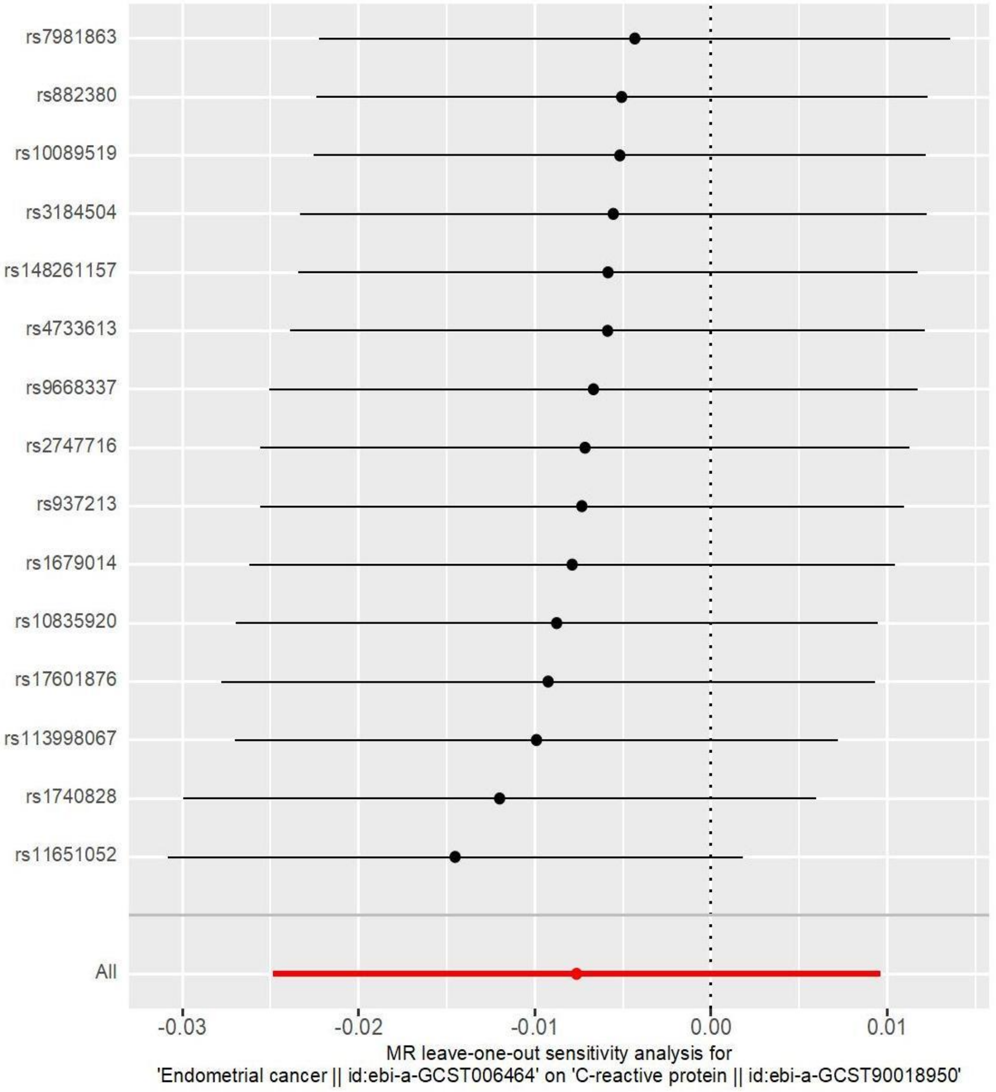

Supplementary Figure S9 Leave-one-out inverse-variance weighted mendelian randomization analysis of endometrioid histology cancer on C-reaction protein

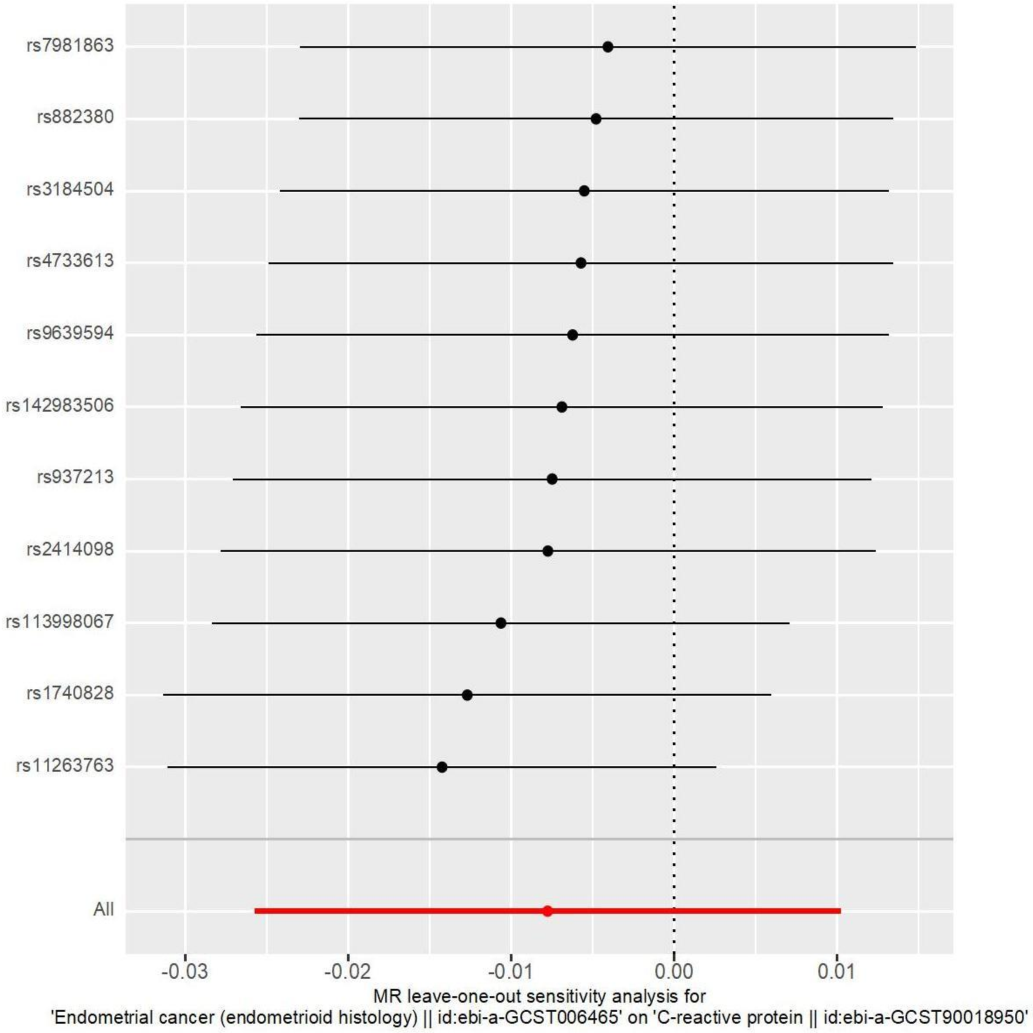

Supplementary Figure S10 Leave-one-out inverse-variance weighted mendelian randomization analysis of non-endometrioid histology cancer on C-reaction protein

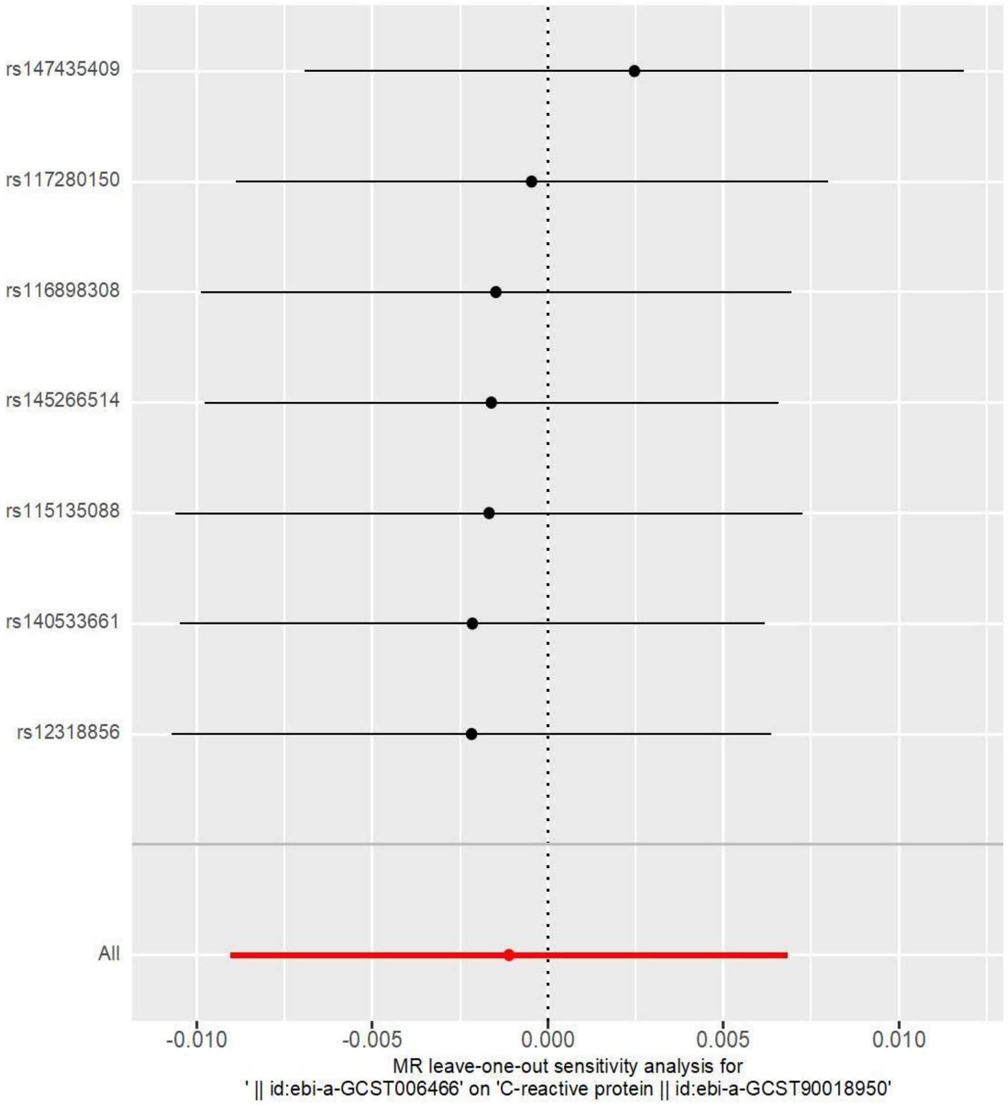

Supplementary Tables

Supplementary Table S1 The demographic profile of the study participants.

Abbreviations: SNPs, single nucleotide polymorphisms; EC, endometrial cancer, EEC, endometrioid histology cancer, NEC, Non-endometrioid histology cancer.

Supplementary Table S1

Traits	PMID	Number of SNPs	Number of cases	Number of controls	Year	Database ID
CRP	34594039	19057467	353466		2021	ebi-a-GCST90018950
EC	30093612	9470555	12906	108979	2018	ebi-a-GCST006464
EEC	30093612	9464330	8758	46126	2018	ebi-a-GCST006465
NEC	30093612	8974630	1230	35447	2018	ebi-a-GCST006466

Supplementary Table S2 The basic characteristics and detailed information of 188 SNPs assocciatied with CRP.

Abbreviations: Chr, chromosome; SNP, single nucleotide polymorphism; EAF, effect allele frequency; SE, standard error, EA, Effect Allele, OA, Other Allele.

Supplementary Table S2

SNP	Chr	Position	EA	OA	EAF	Beta	SE	P value
rs10175899	2	113801150	A	T	0.688408	0.0154	0.0023	3.31E-11
rs1037170	17	72702914	T	C	0.682071	0.0251	0.0024	1.61E-26
rs10493373	1	65412940	G	T	0.85761	-0.0261	0.0034	3.92E-14
rs10629382	19	45253542	CTTTG	C	0.400615	0.0327	0.0025	2.11E-39
rs10644928	11	59960726	CTT	C	0.393705	-0.0191	0.0025	1.28E-14
rs1064725	19	45422561	G	T	0.040721	0.0511	0.0063	5.94E-16
rs10732335	9	15862077	C	A	0.443834	-0.0139	0.0024	1.05E-08
rs10831676	11	11820449	C	A	0.52331	-0.0122	0.0022	4.52E-08
rs11039148	11	47274126	A	T	0.154873	-0.0333	0.0033	1.56E-23
rs11078597	17	1618363	C	T	0.189012	-0.0187	0.0027	6.37E-12
rs11118625	1	221103388	G	A	0.231939	-0.0206	0.0026	3.74E-15
rs112303588	12	121706242	C	G	0.0334301	0.0481	0.0072	2.36E-11
rs11230768	11	61430471	A	G	0.268401	0.0148	0.0025	5.54E-09
rs112875651	8	126506694	A	G	0.350504	-0.0201	0.0023	3.89E-18
rs113040517	19	49162498	C	G	0.763448	0.0184	0.0029	1.66E-10
rs115478735	9	136149711	T	A	0.158589	0.0316	0.003	5.05E-26
rs11577023	1	222061973	C	T	0.309517	-0.016	0.0026	1.02E-09
rs116269315	5	35895782	G	A	0.0250674	0.0422	0.0077	3.68E-08
rs11644809	16	29935505	A	G	0.559117	0.0144	0.0024	3.82E-09
rs11708067	3	123065778	G	A	0.198778	0.0163	0.0028	5.87E-09
rs117211511	12	121701433	A	G	0.0435784	0.0362	0.006	1.73E-09
rs11738559	5	150476129	T	C	0.171287	0.0184	0.0029	2.25E-10
rs11778726	8	126338171	A	G	0.5449	0.021	0.0024	4.19E-18
rs11786900	8	9239958	C	G	0.0971078	-0.0365	0.0041	2.41E-19
rs11812460	10	94832128	A	G	0.332359	-0.014	0.0024	7.12E-09
rs12127241	1	66112676	G	T	0.368713	-0.1263	0.0025	1.00E-200
rs12132412	1	21820042	G	A	0.351357	0.0145	0.0023	4.91E-10
rs12184664	13	42583977	G	A	0.479001	-0.0129	0.0022	5.01E-09
rs1223815	1	214340157	T	C	0.821354	-0.0175	0.0028	4.31E-10
rs12300845	12	24195798	T	G	0.0327159	-0.058	0.0067	8.26E-18
rs12482948	21	17610852	T	C	0.611054	-0.0133	0.0023	5.39E-09
rs12540285	7	150312474	G	A	0.21675	-0.0168	0.0026	1.16E-10
rs1260326	2	27730940	C	T	0.575385	-0.0636	0.0022	3.85E-187
rs12701278	7	1029549	C	T	0.216847	-0.0237	0.0029	7.11E-16
rs12713422	2	25134497	C	A	0.453118	0.0186	0.0024	1.49E-14
rs12730935	1	154419892	A	G	0.406094	-0.0827	0.0022	1.00E-200

rs1292067	17	57930646	T	G	0.391587	-0.0185	0.0022	4.69E-17
rs12944581	17	76348830	C	G	0.71924	0.0272	0.0025	4.62E-28
rs12952093	17	76363640	C	A	0.730439	-0.032	0.0028	1.31E-30
rs12992747	2	178190491	C	A	0.811195	-0.0164	0.0028	6.94E-09
rs12992995	2	175197545	A	C	0.237371	-0.0154	0.0026	3.08E-09
rs13013	10	75562161	A	C	0.488783	0.0147	0.0023	4.23E-10
rs13084243	3	49821878	C	G	0.323847	0.0228	0.0026	7.12E-19
rs13242809	7	22746564	A	T	0.360543	-0.0295	0.0024	2.87E-34
rs1338071	6	153365834	C	G	0.3381	0.0157	0.0023	1.71E-11
rs1348675	13	58712638	A	G	0.217104	0.0179	0.0026	1.27E-11
rs138335	22	41227086	G	C	0.618233	0.0147	0.0022	5.79E-11
rs141179989	16	51067084	T	C	0.0170166	-0.0552	0.01	3.90E-08
rs1412444	10	91002927	T	C	0.317982	0.0237	0.0023	1.14E-24
rs141783576	6	127439897	C	G	0.0730835	-0.0444	0.0047	4.32E-21
rs1441169	2	214033530	G	A	0.524741	-0.0238	0.0024	6.14E-23
rs146902872	5	156700886	C	T	0.039272	-0.0341	0.0062	3.97E-08
rs1470559	3	35670105	A	G	0.324111	0.0133	0.0023	1.36E-08
rs1477890	4	18511738	G	A	0.48824	0.0137	0.0021	1.62E-10
rs147842318	9	102248825	C	T	0.352685	0.0147	0.0025	8.37E-09
rs1490384	6	126851160	T	C	0.592164	-0.0307	0.0024	1.76E-37
rs149487415	20	44585054	G	A	0.0546215	-0.0351	0.0053	4.79E-11
rs149624078	15	53728710	T	C	0.0139395	-0.1301	0.0105	3.42E-35
rs150844304	15	43726625	C	A	0.0245091	0.0834	0.0078	7.59E-27
rs1545536	8	144643169	T	C	0.256588	-0.0158	0.0025	2.67E-10
rs1546721	6	109625796	C	T	0.393537	-0.0122	0.0022	2.99E-08
rs17088032	4	68003444	A	T	0.165794	0.0179	0.0032	3.10E-08
rs17138478	17	36073320	A	C	0.144262	0.0318	0.003	2.14E-25
rs17616063	16	51436882	G	A	0.076093	-0.1262	0.0045	1.78E-170
rs17652767	15	53169284	A	G	0.115538	-0.0237	0.0033	1.37E-12
rs178795	17	16123873	A	G	0.438631	0.0139	0.0023	2.53E-09
rs1800693	12	6440009	C	T	0.359228	-0.0194	0.0023	1.40E-17
rs1800961	20	43042364	T	C	0.027432	-0.1005	0.0066	3.55E-52
rs1800973	12	69744014	A	C	0.0611841	0.0308	0.005	8.81E-10
rs191597741	1	21380368	A	G	0.416675	-0.0133	0.0024	4.97E-08
rs1933737	6	116310287	C	T	0.284843	-0.017	0.0025	6.93E-12
rs2049045	11	27694241	C	G	0.186853	-0.0214	0.0031	3.92E-12
rs2075184	2	103080592	C	T	0.736527	-0.0183	0.0025	1.36E-13
rs2110944	2	37090233	C	T	0.502958	0.0144	0.0022	3.68E-11
rs2115885	5	87598818	A	G	0.204417	-0.0173	0.003	9.06E-09
rs2161037	2	169893419	A	G	0.57616	0.0203	0.0022	3.87E-20
rs2161374	5	172176886	T	C	0.471773	-0.0156	0.0021	4.41E-13
rs2211320	1	159693605	A	G	0.329673	-0.1724	0.0025	1.00E-200
rs2239222	14	73011885	G	A	0.361299	0.0301	0.0022	3.63E-41
rs2250010	12	47193818	T	C	0.765915	0.0177	0.0026	6.44E-12
rs2303998	12	121655063	A	G	0.0155883	-0.0485	0.0087	2.74E-08
rs2306881	3	4753712	G	A	0.432899	-0.0138	0.0023	1.99E-09
rs2393794	12	121378976	C	T	0.166708	0.033	0.0029	2.79E-29
rs2424698	20	25256106	T	C	0.527889	0.0153	0.0023	4.95E-11
rs2428953	1	44443459	A	G	0.101123	0.0199	0.0035	2.04E-08
rs2710804	7	36084529	C	T	0.340047	0.0193	0.0023	4.43E-17
rs2818392	10	133946337	G	A	0.733282	-0.0142	0.0026	2.93E-08
rs2836881	21	40466299	T	G	0.2463	-0.0295	0.0025	6.49E-32
rs28429148	16	53798319	A	G	0.408559	0.0164	0.0022	1.94E-13
rs2844485	6	31534206	G	A	0.616638	0.0161	0.0025	8.63E-11
rs288183	5	107348180	G	T	0.207083	-0.0158	0.0027	3.57E-09
rs28929474	14	94844947	T	C	0.0202948	-0.1027	0.0085	2.27E-33
rs2933235	12	56921418	G	A	0.166135	-0.0228	0.0032	1.56E-12
rs2961108	8	103511148	A	G	0.143171	0.0197	0.0031	2.45E-10
rs2972558	19	45356141	T	C	0.590593	-0.0437	0.0023	2.18E-78
rs3115574	6	32217413	C	T	0.627324	0.0272	0.0025	7.49E-28
rs33951980	7	73029437	T	C	0.125343	-0.0301	0.0032	1.51E-20
rs340023	15	60908082	T	C	0.361219	0.019	0.0024	7.68E-15

rs351978	19	806256	G	A	0.561368	-0.0127	0.0022	1.68E-08
rs35308741	2	203092677	G	A	0.710524	-0.0155	0.0028	2.81E-08
rs35440896	2	61496141	AT	A	0.441498	-0.0134	0.0022	1.31E-09
rs371271858	9	104113739	T	A	0.158104	0.0219	0.0033	3.56E-11
rs3752442	4	3446883	G	A	0.28575	0.0163	0.0024	1.52E-11
rs3768321	1	40035928	T	G	0.188308	0.0282	0.0028	1.21E-24
rs3843540	7	99126640	C	T	0.179492	-0.0245	0.0028	3.44E-18
rs3935032	1	1564194	T	C	0.377302	-0.0178	0.0025	1.55E-12
rs4018180	16	2169458	A	G	0.0674583	-0.0314	0.0048	8.10E-11
rs4237643	11	43648368	G	T	0.72519	-0.0163	0.0024	3.01E-11
rs429358	19	45411941	C	T	0.145853	-0.2447	0.003	1.00E-200
rs4304577	1	154352133	C	T	0.154218	-0.0557	0.0033	1.22E-62
rs4330804	9	139294247	G	T	0.454183	0.0179	0.0024	1.10E-13
rs4389587	4	38211434	A	C	0.757408	-0.0144	0.0026	1.92E-08
rs4426089	10	62223112	T	C	0.425148	-0.0119	0.0022	4.73E-08
rs4433388	1	159729337	G	C	0.661377	0.0419	0.0024	3.26E-70
rs45582641	2	113954629	A	G	0.0709905	-0.0255	0.0042	1.90E-09
rs4640929	6	7004861	A	G	0.491977	0.0158	0.0024	1.14E-10
rs4655802	1	65888231	A	G	0.639209	-0.022	0.0023	1.15E-21
rs469864	1	91542517	C	T	0.209594	-0.0307	0.0026	3.50E-31
rs4704963	5	158247378	C	T	0.0699101	0.0244	0.0042	7.82E-09
rs4809328	20	62348327	T	C	0.666096	0.0239	0.0025	7.68E-21
rs4821802	22	39037552	G	A	0.352485	-0.0319	0.0025	8.84E-37
rs4839716	6	98400651	C	T	0.399853	-0.0179	0.0025	3.86E-13
rs4842708	12	90428447	T	C	0.616272	-0.0143	0.0023	2.28E-10
rs4851487	2	102302978	T	C	0.405237	0.0139	0.0022	1.77E-10
rs519790	11	72504141	G	C	0.333541	0.0189	0.0023	1.09E-16
rs523288	18	57848369	T	A	0.23446	0.0189	0.0028	2.87E-11
rs5402	3	170727739	A	T	0.120676	0.0255	0.0037	7.06E-12
rs55709272	2	113867288	C	T	0.364095	0.0429	0.0024	2.27E-73
rs55741140	18	60216234	T	C	0.259878	-0.0154	0.0028	2.95E-08
rs55855238	18	55089715	C	T	0.564346	0.0244	0.0023	1.56E-25
rs55880988	16	88546253	A	G	0.347313	0.018	0.0025	1.22E-12
rs56015600	1	247601886	G	A	0.622257	0.0336	0.0022	4.09E-52
rs56246620	1	109816863	TC	T	0.157638	0.0181	0.003	1.53E-09
rs59012264	1	159251511	G	A	0.308688	0.0268	0.0024	1.79E-29
rs6020488	20	49031332	T	C	0.334621	0.0131	0.0023	9.76E-09
rs6089985	20	61377272	T	C	0.316375	-0.0147	0.0025	6.68E-09
rs6090040	20	62692060	C	A	0.552081	-0.0142	0.0022	9.04E-11
rs60987662	7	101844851	G	A	0.322829	0.0137	0.0024	9.68E-09
rs61542988	7	22882291	T	C	0.337956	-0.0206	0.0025	9.36E-17
rs61838755	1	247569423	G	T	0.674087	-0.0187	0.0026	4.72E-13
rs62104180	2	466003	A	G	0.0501154	-0.0325	0.0055	3.96E-09
rs62172792	2	174860672	A	C	0.103207	0.023	0.0039	5.37E-09
rs62193162	2	242268436	A	T	0.416012	0.0157	0.0022	1.88E-12
rs62229260	21	37437566	A	G	0.548319	-0.0137	0.0022	7.17E-10
rs62246311	3	9498143	A	G	0.08219	0.0251	0.004	3.15E-10
rs62618693	11	32956492	T	C	0.0454316	-0.0316	0.0058	4.46E-08
rs645692	17	40701073	T	C	0.287375	0.0185	0.0027	3.57E-12
rs6486122	11	13361524	T	C	0.616462	0.0261	0.0023	3.05E-29
rs653170	1	112328245	T	C	0.370343	0.0127	0.0022	1.10E-08
rs66985888	16	51405303	G	A	0.955792	-0.0348	0.0062	1.95E-08
rs67919109	1	198147819	T	G	0.107628	0.0245	0.0039	2.70E-10
rs6792725	3	24520283	G	A	0.651422	-0.0153	0.0023	5.96E-11
rs687339	3	135932359	T	C	0.781408	-0.0229	0.0026	1.29E-18
rs6920220	6	138006504	A	G	0.180228	0.0203	0.0029	1.76E-12
rs7012637	8	9173209	A	G	0.554123	0.0451	0.0023	1.01E-82
rs7036107	9	92177897	G	A	0.510067	0.0216	0.0025	1.37E-18
rs704017	10	80819132	G	A	0.528942	-0.0159	0.0022	3.50E-13
rs7084062	10	133736636	G	A	0.506386	0.0152	0.0022	4.09E-12
rs7133949	12	95849798	G	T	0.407958	-0.0241	0.0022	8.36E-28
rs71426964	14	73360327	T	C	0.497699	-0.0166	0.0024	4.26E-12
rs7193413	16	28644663	T	A	0.699712	0.0139	0.0024	1.46E-08

rs72694391	14	24874026	C	T	0.402021	-0.0136	0.0023	6.51E-09
rs728538	16	51205819	G	T	0.137816	0.0441	0.0032	3.75E-43
rs72999033	19	19366632	T	C	0.0537871	0.0441	0.0049	1.31E-19
rs73137144	7	74073590	G	A	0.161779	-0.0185	0.003	5.86E-10
rs73351138	14	96931102	C	A	0.317297	-0.0154	0.0024	7.31E-11
rs74154640	1	247596860	A	C	0.160756	-0.0238	0.0029	4.45E-16
rs746839	8	142617261	G	C	0.331698	0.0133	0.0024	2.33E-08
rs7485554	12	84070366	A	G	0.572216	0.0139	0.0025	3.22E-08
rs74904971	4	89050026	A	C	0.148655	-0.019	0.003	4.37E-10
rs7511796	1	236305250	A	T	0.619708	-0.0155	0.0025	4.75E-10
rs75460349	1	27180088	C	A	0.0236047	-0.0934	0.008	3.82E-31
rs75690706	3	101815428	G	T	0.100469	0.0248	0.004	7.91E-10
rs7662792	4	45121873	T	A	0.645771	0.0129	0.0022	9.51E-09
rs7673508	4	154769597	T	C	0.409241	-0.0123	0.0022	4.16E-08
rs77216612	12	12877983	G	A	0.310048	0.0138	0.0023	3.20E-09
rs77704739	5	52080909	C	T	0.0344075	-0.0511	0.006	1.22E-17
rs7828742	8	116960729	G	A	0.595624	0.0225	0.0022	8.70E-25
rs7979473	12	121420260	G	A	0.5983	0.1272	0.0022	1.00E-200
rs8051062	16	69551473	C	T	0.559122	-0.0162	0.0022	2.45E-13
rs8060025	16	27327214	G	T	0.545205	-0.0156	0.0022	3.13E-12
rs8178824	17	64224775	T	C	0.0297335	0.0582	0.0071	2.75E-16
rs832578	5	56164223	T	C	0.827135	0.0187	0.0032	4.40E-09
rs9366639	6	26167613	G	C	0.155422	-0.0281	0.003	7.40E-21
rs939584	2	621558	T	C	0.841583	0.0167	0.0029	1.45E-08
rs9521499	13	110393185	C	T	0.462515	0.0128	0.0022	6.44E-09
rs9604045	13	113927208	T	G	0.232373	-0.0193	0.0027	9.15E-13
rs9738365	12	31997635	A	T	0.253209	0.0168	0.0025	1.36E-11

Supplementary Table S3 Causal associations of C-reaction protein with endometrial cancer and its subtypes in the forward MR analysis.

Abbreviations: IVW, inverse variance-weighted, OR:odds ratio, CI: confidence interval, MR: Mendelian randomization.

Supplementary Table S3

Methods	Endometrial cancer		Endometrial histology		Non-endometrial histology	
	OR (95%CI)	*P*	OR (95%CI)	*P*	OR (95%CI)	*P*
IVW	1.097(0.996-1.208)	0.061	1.110(1.000-1.231)	0.049	1.250(1.005-1.555)	0.045
MR-Egger	1.009(0.881-1.156)	0.892	1.040(0.899-1.203)	0.599	1.397(1.028-1.899)	0.034
Weight Median	0.998(0.866-1.128)	0.862	1.023(0.872-1.201)	0.778	1.596(1.084-2.350)	0.018
Weight Mode	1.028(0.935-1.131)	0.572	1.044(0.925-1.180)	0.485	1.282(0.976-1.682)	0.075

Supplementary Table S4 Causal associations of endometrial cancer and its subtypes with C-reaction protein in the reverse MR analysis.

Abbreviations: IVW, inverse variance-weighted, OR: odds ratio, CI: confidence interval, MR: Mendelian randomization.

Supplementary Table S4

Methods	Endometrial cancer		Endometrial histology		Non-endometrial histology	
	OR (95%CI)	*P*	OR (95%CI)	*P*	OR (95%CI)	*P*
IVW	0.99(0.98-1.01)	0.39	0.99(0.97-1.01)	0.40	1.00(0.99-1.01)	0.78
MR-Egger	1.03(0.96-1.11)	0.37	1.06(0.97-1.16)	0.25	0.99(0.97-1.01)	0.57
Weight Median	0.99(0.97-1.01)	0.28	0.99(0.97-1.01)	0.17	1.00(0.99-1.01)	0.84
Weight Mode	1.02(0.97-1.06)	0.53	0.97(0.94-1.01)	0.18	1.00(0.99-1.02)	0.68
